# Heads or Tails: Do Stranded Fish (Mosquitofish, *Gambusia affinis*) Know Where They Are on a Slope and How to Return to the Water?

**DOI:** 10.1371/journal.pone.0104569

**Published:** 2014-08-27

**Authors:** Robert J. Boumis, Lara A. Ferry, Cinnamon M. Pace, Alice C. Gibb

**Affiliations:** 1 Department of Biology, Northern Arizona University, Flagstaff, Arizona, United States of America; 2 School of Mathematical and Natural Sciences, Arizona State University, Glendale, Arizona, United States of America; University of Utah, United States of America

## Abstract

Aquatic vertebrates that emerge onto land to spawn, feed, or evade aquatic predators must return to the water to avoid dehydration or asphyxiation. How do such aquatic organisms determine their location on land? Do particular behaviors facilitate a safe return to the aquatic realm? In this study, we asked: will fully-aquatic mosquitofish (*Gambusia affinis*) stranded on a slope modulate locomotor behavior according to body position to facilitate movement back into the water? To address this question, mosquitofish (n = 53) were placed in four positions relative to an artificial slope (30° inclination) and their responses to stranding were recorded, categorized, and quantified. We found that mosquitofish may remain immobile for up to three minutes after being stranded and then initiate either a “roll” or a “leap”. During a roll, mass is destabilized to trigger a downslope tumble; during a leap, the fish jumps up, above the substrate. When mosquitofish are oriented with the long axis of the body at 90° to the slope, they almost always (97%) initiate a roll. A roll is an energetically inexpensive way to move back into the water from a cross-slope body orientation because potential energy is converted back into kinetic energy. When placed with their heads toward the apex of the slope, most mosquitofish (>50%) produce a tail-flip jump to leap into ballistic flight. Because a tail-flip generates a caudually-oriented flight trajectory, this locomotor movement will effectively propel a fish downhill when the head is oriented up-slope. However, because the mass of the body is elevated against gravity, leaps require more mechanical work than rolls. We suggest that mosquitofish use the otolith-vestibular system to sense body position and generate a behavior that is “matched” to their orientation on a slope, thereby increasing the probability of a safe return to the water, relative to the energy expended.

## Introduction

Some fully-aquatic fishes routinely leap out of the water [1] and strand themselves on land to avoid aquatic predators [2] or other inhospitable conditions [3]. However, a stranded fish faces a series of challenges when attempting to return to the water. First, the fish must determine its position on land, relative to the aquatic environment. Second, the fish must produce an effective locomotor behavior – that is, it must produce body movements that generate a net displacement of the fish away from its starting position. Third, these locomotor movements must ultimately propel the fish back into the water. If non-airbreathing fishes cannot return to the water, they risk dying from asphyxiation, desiccation, or both [4].

Individuals of *Gambusia affinis,* the mosquitofish (a member of the Teleostei: Cyprinodontiformes, the toothed “carps”), are considered fully-aquatic fishes. However, individuals are known to voluntarily strand themselves on banks and emergent vegetation in an effort to evade predatory fishes [2]. Stranded mosquitofish will quickly return to the water, but the behavior that they use to move over land and return to the aquatic realm has yet to be described. In the laboratory environment, mosquitofish will produce a “tail-flip” jump when manually stranded on a flat surface, i.e. experimental arena with 0% grade [5]. However, in the wild, fish leaving the water are likely to land on a slope – such as a creek bank or beach – where a variety of factors could influence the behavioral response to stranding and the ultimate outcome of a fish’s attempt to return to the water. Such factors include: the initial orientation of the body of the fish relative to the slope, how quickly the fish responds to being stranded, and the nature of the movements produced by the fish in response to stranding.

In this study, individual mosquitofish were involuntarily stranded in the laboratory on an artificial slope designed to simulate a stream bank and the behavioral response to stranding was recorded with a digital camcorder. By systematically varying the orientation of the fish’s body relative to the slope, we asked: is the behavioral response to stranding related to initial body position, and does body position affect the ability of a fish to move downslope? The behavioral responses to stranding were categorized based on the appearance of stereotyped movement patterns and then compared by quantifying performance variables, including success in reaching the water. We used these findings to address the overarching question: can stranded fish recognize their body orientation relative to a slope and ‘tune’ their locomotor behavior to that orientation to move back into the water effectively and efficiently?

## Materials and Methods

### Ethics statement

All care/handling procedures and experiments for this study were approved under NAU IACUC Protocol #09-009. Mosquitofish, *Gambusia affinis* (Baird and Girard 1853), were obtained by Arizona Game and Fish employees from ponds in Arizona (USA) and held in the laboratory at Northern Arizona University in two 75-liter tanks; each tank was provided with filtration, aeration, and a 12∶12 light:dark cycle. At the end of the experiments, all fish were euthanized with an overdose of buffered tricaine methanesulfonate (following AVMA Guidelines on Euthanasia) so that each individual could be used for anatomical studies.

### Experimental trials

**Figure 1:**
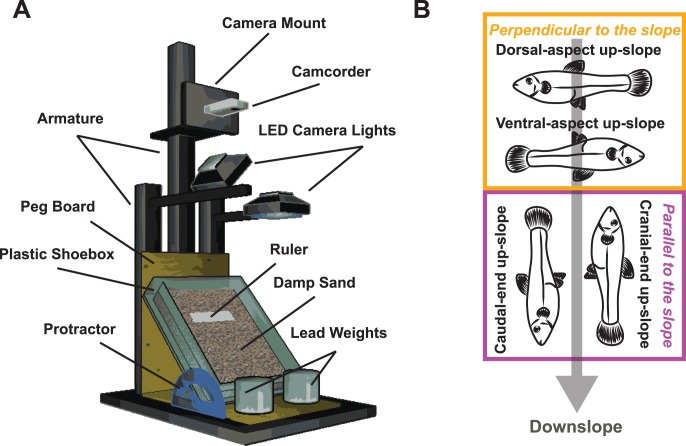
An artificial slope apparatus was used to examine the response of individual mosquitofish (*Gambusia affinis*) to being stranded on land. (A) A photography armature held a digital camcorder and commercial-grade LED lights directly over a plastic box that was filled with damp sand and positioned at 30° inclination. Fish were placed on the damp sand and the response to stranding was recorded with the digital camera to a flash memory card. (B) Four initial body positions were used in the slope trials; for two of these positions, the long axis of the body was oriented perpendicular (at 90°) to the slope (dorsal-aspect up-slope and ventral-aspect up-slope); for the other two positions, the long axis of the body was oriented parallel to the slope (cranial-aspect up-slope and caudal-aspect up-slope). One trial at a randomly assigned body position was conducted for each individual included in the study, with a minimum of n = 9 for a given position and a total n = 53; see text for details.

The artificial slope apparatus used in the experimental trials consisted of a photography armature (three arms attached to a fixed base), a 24×30 cm plastic box filled with damp sand, a consumer-grade digital camcorder (HD 720p with an SD flash card), and two LED-array commercial photography lights (Figure 1). The slope apparatus was assembled with the camcorder and photography lights were mounted on the armature and oriented over the box. The plastic box was positioned at an angle to create a slope and individual mosquitofish were manually placed in a target area at the apex of the slope during the experimental trials.

Wet sand was used in the experiments because it emulated a natural substrate and was neither too adherent (“sticky”) nor too lubricous (“slippery”). Using two dead fish weighing 0.73 g and 1.76 g (the approximate size range of the individuals used in the experimental trials) the angle of repose (the angle at which an inert fish would remain in place without slipping or rolling off) for a mosquitofish resting on wetted sand was determined to be between 40° and 45°. Based on this finding, we used an experimental slope of 30°, with the objective of providing mosquitofish with both a strong sensory stimulus and a grade that required active movement by the fish to move downhill. Because preliminary trials indicated that the physical position of the slope apparatus with in our laboratory had no effect on behavior, all experimental trials were carried out with the experimental apparatus in a single compass orientation and physical location.

During the experimental trials, mosquitofish individuals (total n = 53, with 42 females and 11 males; see Appendix S1) were artificially stranded by manually placing fish within in a 7.5×7.5 cm “target” area located at the apex of the slope and the response to stranding was recorded using the digital camcorder (see Video S1, Video S2, Video S3 and Video S4). For all of these experiments, a single trial was recorded for an individual and the mosquitofish was euthanized immediately after the trial. The first set of experimental trials was conducted by placing female mosquitofish (n = 42) in one of four *body positions*: cranial end up-slope, caudal end up-slope, dorsal aspect up-slope, or ventral aspect upslope (Figure 1). During these trials, initial body position for each female mosquitofish was determined using a random number generator, until we reached a minimum of nine trials for a given body position. In a second set of trials all male fish (n = 11) were placed in the dorsal-aspect up-slope position and then compared with female fish placed in the same body position. The four body positions used for the stranding trials could also be considered as two *body orientations*. Cranial-end and caudal-end up-slope were considered *parallel* to the slope because the long axis of the fish’s body followed the slope. Dorsal-aspect and ventral-aspect up-slope were considered *perpendicular* because the long axis of the fish’s body was at 90° to the slope.

Video sequences were deconstructed into individual image files and fish movements quantified with digitizing software, specifically ImageJ, developed by W.S. Rasband at the National Institute of Health and Didge, developed by A. J. Cullum at Creighton University. Using the deconstructed image sequences, the behavioral response to stranding was categorized and quantified to produce a series of response variables (data for all 53 individuals are given in Appendix 1). *Latency time (s)* is the time a fish remained motionless after being positioned on the sand within the target area. *Landing time (s)* is the total duration of the behavior, and was measured as the time from the onset of the response until the time the fish stopped moving. *Response trajectory*, or overall angle of travel, was determined by the landing position of the fish relative to the starting position, with 0° indicating the fish moved directly down-slope. To evaluate the effectiveness of a particular behavioral response to stranding, the *outcome* of the initial (first) movement of an individual fish was categorized as either a success or a failure. The outcome of a trial was deemed a “success” if the fish reached the bottom of the experimental arena as a result of its initial movement in response to stranding. The outcome was deemed a “failure” if the fish did not reach the bottom of the arena. The data supporting the results of this article are included as Appendix 1 and in Videos S1, S2, S3, and S4.

### Analyses

Two timing variables (latency and landing time) were examined for potential monotonic associations with body mass and standard length. For this analysis we used Spearman rank correlation, a one-tailed *a priori* hypothesis that small fish are faster than large fish, and a null hypothesis of no change in timing variables with body size. All Spearman rank correlations of size vs. timing were performed with SPSS (v. 21 for OSX).

To assess potential differences in the behavioral response to stranding between the two sexes, males were compared to females in the dorsal aspect up-slope starting position, as described previously. In this analysis, we considered the possibility that males and females could differ in the movement class they produced in response to stranding (leaps vs. rolls), and/or in movement outcome (success vs. failure). Fisher’s Exact tests were used to ascertain if there are nonrandom associations between sex and either of these two categorical variables (sex vs. movement class and sex vs. movement outcome), with the null hypothesis that the distribution of responses for the response variables is the same for males as it is for females. All Fisher’s Exact tests were performed using Graphpad QuickCals. Once male responses were determined to be statistically indistinguishable from female responses (see Results), the data were pooled for subsequent analyses.

Using the pooled male and female datasets, a series of Fisher’s Exact tests was used to evaluate potential nonrandom associations between three categorical variables: body orientation (independent), movement class (dependent), and movement outcome (dependent). To test the hypothesis that fish vary behavior according to initial body orientation, we used a Fisher’s Exact test to examine which movement class (leap vs. roll) was produced in response to different body orientations (parallel vs. perpendicular); the null hypothesis was that leaps and rolls are equally likely to occur in either body orientation. A second Fisher’s Exact test was used to determine if initial body orientation (parallel vs. perpendicular) affected movement outcome (success vs. failure), with the null hypothesis that successes and failures are equally likely to occur in either body orientation. A third Fisher’s Exact test was used to determine if the movement class produced in response to stranding (leap vs. roll) affected movement outcome (success vs. failure), with the null hypothesis that successes and failures are equally likely to occur in both movement classes. Subsequently, we pooled all the behavioral responses to stranding and used a *G*-test to examine the *a posteriori* hypothesis that, when all responses are considered together, successes occur more often than failures.

We also sought to determine if response trajectory (the angle of movement downslope) varied according to either initial body orientation (parallel vs. perpendicular) or movement class (leaps vs. rolls). For this analysis, we employed Welch’s tests to accommodate unequal sample sizes and heterogeneous variances within the data. All tests of normality, homogeneity of variances (Levene’s test), and Welch’s tests were conducted using SPSS (v. 21 for OSX). In these analyses, two Welch’s tests were used consider if either body orientation or movement class affected response trajectory. The null hypothesis for the Welch’s tests was that there is no effect of either categorical variable on the direction of downslope movement.

## Results

### Behavioral response to stranding

**Figure 2:**
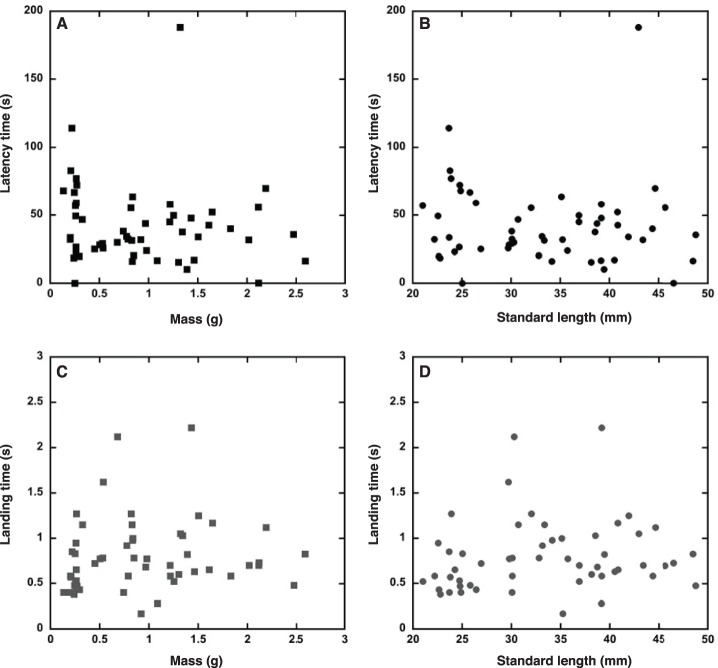
There is little effect of body size on latency and landing times for mosquitofish (*Gambusia affinis*) stranded on a slope. There was no monotonic relationship between body size and the time to respond to stranding as measured by latency time: (A) body mass vs. latency time and (B) standard length vs. latency time. There was a weak monotonic relationship between body size and the total duration of the movement produced in response to stranding as measured by landing time: (C) body mass vs. landing time and (D) standard length vs. landing time. Relationships between size and timing were assessed using Spearman’s rank correlation analysis under the *a priori* hypothesis that larger fish are slower and the null hypothesis of no change in timing with size; see text for additional details.

After being manually stranded on an artificial slope in one of four body positions (Figure 1), mosquitofish often remained motionless for 40+ seconds (mean ± SEM of 42±4 s) before they began to move (Figure 2; see also Video S1, Video S2, Video S3 and Video S4). This extended response latency suggests that the movement produced by mosquitofish in response to stranding is a deliberate movement, and not merely reflexive thrashing produced in reaction to exposure to air. When mosquitofish did finally move, the resulting behavior generated rapid progress downslope, and fish typically stopped moving within a second (0.8±0.05 s) of initiating movement (Figure 2).

**Figure 3:**
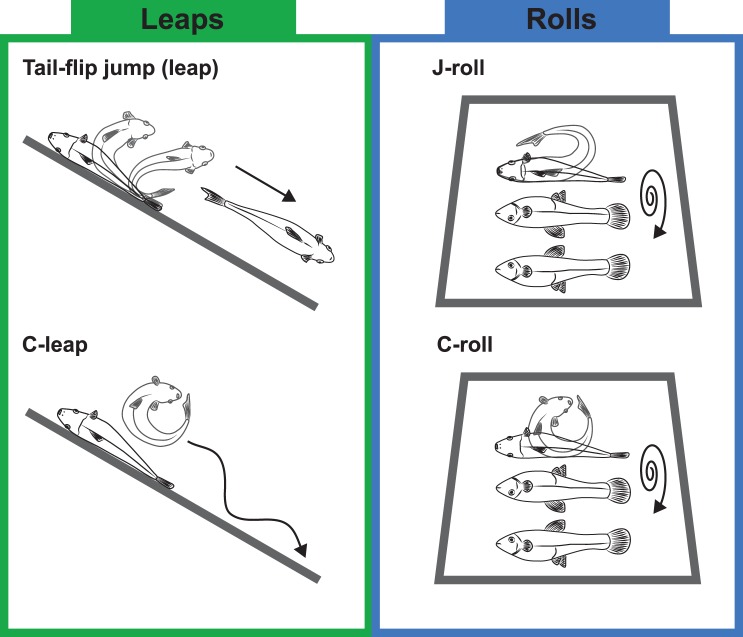
Mosquitofish (*Gambusia affinis*) produced four distinct behavioral responses to being stranded on a slope: tail-flip jumps (a type of leap), C-leaps, J-rolls, and C-rolls. During leaps (tail-flip jumps and C-leaps), mosquitofish produced sufficient momentum to elevate the body above the substrate. During rolls (C-rolls and J-rolls), axial body movements destabilized the fish’s mass, enabling the fish to tumble downslope.

Of the 53 experimental stranding trials conducted (n = 53 individual mosquitofish, with one trial per individual; see Appendix S1), two individuals exhibited behaviors that could not be categorized based on stereotypical movement patterns. However, the other 51 responses could be readily categorized as one of four locomotor behaviors (Figure 3). Two of these behaviors have been described in previous studies, but basic descriptions are included here to facilitate comparison with the two newly described behaviors. During a *tail-flip jump* [5], a mosquitofish leaps from the ground via a two-stage propulsive movement. During the first stage, the fish curls its anterior region away from the substrate to accelerate the anterior body and center of mass first vertically, and then caudally (Figure 3; Video S1). During the second stage, the fish straightens the body and pushes off the substrate using the caudal peduncle (posterior body and tail fin); through this extension of the body the fish leaps from the substrate and into a caudally-directed, ballistic flight path [5]. During a *C-leap,* a fish typically lifts the cranial and caudal body regions up away from the substrate (although initial bending toward the substrate can also occur [6]); as a result of this movement, momentum is transferred to the center of mass and the body of the fish is propelled vertically, up above the substrate (Figure 3; Video S2). After landing, some fish generated additional side-to-side bending movements that appeared to aid in producing prolonged downslope movement.

Two previously undescribed behaviors were also observed during the study. A *C-roll* appears to be similar to, but less forceful than, a C-leap. During a C-roll (Figure 3; Video S3), the combined movements of the cranial and caudal regions are not forceful enough to propel the fish into the air, but instead destabilize the center of mass to initiate a downhill tumble in which the fish’s body remains in contact with the substrate throughout the roll. During a *J-roll*, the fish bends the caudal region of the body (posterior body plus tail fin) up toward the cranial (head) end of the body (Figure 3; Video S4); this motion also triggers a down-slope tumble where the fish’s body remains in contact with the substrate.

**Figure 4:**
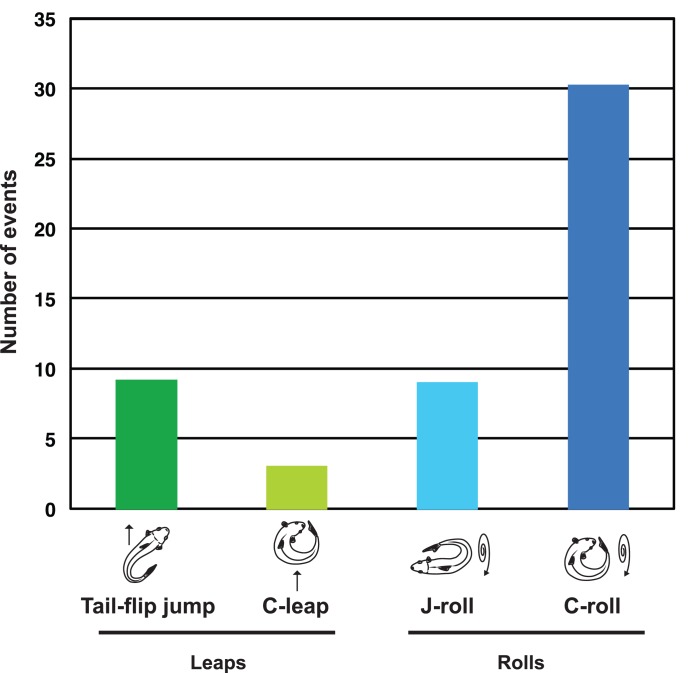
During the experimental trials, most (39 out of 51) mosquitofish (Gambusia affinis) rolled downslope in response to stranding. Columns represent the number of responses recorded for a given behavior: tail-flip jumps (a type of leap, n = 9), C-leaps (n = 3), J-rolls (n = 9), and C-rolls (n = 30).

After these four responses to stranding were identified, they were then assigned to two movement classes based on similar physical demands of the behaviors. Behaviors where the center of mass is vaulted up above the substrate (tail-flip jumps and C-leaps) were categorized as leaps. Behaviors where the body remains in contact with the ground (C-rolls and J-rolls) were categorized as rolls. During the experiments, mosquitofish produced more rolls than leaps, and C-rolls were the most commonly produced behavior overall (30 out of 51 responses; Figure 4).

### Body Size

We initially expected that small fish would be faster than large fish (a one-tailed hypothesis) due to well-established allometric (scaling) effects on the duration of locomotor behaviors [7]. However, for mosquitofish stranded in the laboratory (Figure 2), there was no effect of size on response latency and little effect on duration of the behavior (measured here as landing time). There was no monotonic relationship between mass and latency time (Spearman’s rho =  −0.141, n = 53, *p* = 0.313), and only a weak relationship between mass and landing time (Spearman’s rho = 0.264, n = 53, *p* = 0.057). Similarly, there was no monotonic relationship between standard length and latency time (Spearman’s rho =  −0.096, n = 53, *p* = 0.492), and only a weak association between standard length and landing time (Spearman’s rho = 0.287, n = 53, *p* = 0.037). Small fish tended to come to a halt more quickly than larger fish, presumably because they have less inertia and proportionally greater contact between skin and substrate, which will generate frictional forces that decelerate the fish. However, there is considerable variability in landing time across all body sizes (Figure 2).

### Sex

**Figure 5:**
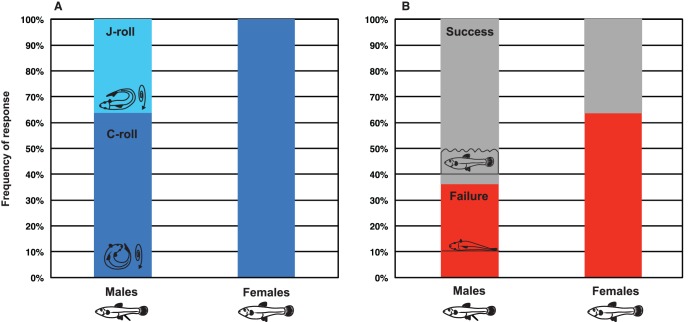
Male (n = 11) and female (n = 11) mosquitofish (*Gambusia affinis*) produced only rolls (and never leaps) in response to being stranded with the dorsal aspect of the body oriented toward the top of the slope (Fisher’s Exact test df = 1, n = 21, *p* = 1); however, male fish produced two types of rolls, whereas females only produced C-rolls. Although females tended to fail to reach the bottom of the arena more often than males (B), males and females were not statistically different in this ability (Fisher’s Exact test df = 1, n = 22, *p* = 0.395).

Because there was only a small sample size available for male mosquitofish in our experimental population (male n = 11, female n = 42, see Methods), all male individuals were placed in one initial body position and compared with females placed in the same position (dorsal-aspect up-upslope, see Figure 1). In terms of both the movement class produced in response to stranding (leaps vs. rolls) and in overall movement outcome (i.e., ability to reach the bottom of the slope, considered here as success, or inability to reach the bottom of the slope, or failure), there was no difference in the male and female response to stranding (Figure 5A). Both males and females only produced rolls (and never leaps) in response to being stranded with their dorsal aspect of the body oriented toward the top of the slope (Fisher’s Exact test df = 1, n = 21, *p* = 1): however, male fish produced both types of rolls, whereas females only produced C-rolls. Although females tended to fail to reach the bottom of the arena more often than males, males and females were not statistically different in their ability to successfully reach the bottom (Fisher’s Exact test df = 1, n = 22, *p* = 0.395; Figure 5B). Because there were no statistically significant differences in the male and female responses to stranding, the data sets were subsequently pooled to create a composite dataset that contained the responses of both sexes.

### Body Orientation, Movement Class, and Movement Outcome

**Figure 6:**
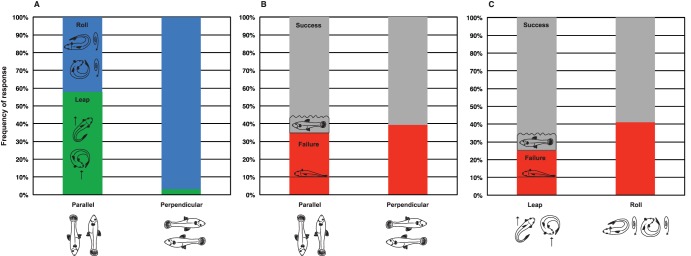
Initial body orientation was associated with the production of certain behaviors (leaps vs. rolls) when mosquitofish (*Gambusia affinis*) were stranded on a slope, but movement outcome (success vs. failure) was independent of body orientation and movement class. (A) When placed with the body’s long axis perpendicular (at 90°) to the slope (n = 32), mosquitofish were more likely to produce a roll than a leap (Fisher’s Exact test, df = 1, n = 51, *p* = 0.00002); in contrast, when mosquitofish were placed with the body’s long axis parallel to the slope (n = 19), rolls and leaps occurred at approximately the same frequency. (B) The probability of successfully moving downslope was similar from either initial body orientation (Fisher’s Exact test, df = 1, n = 53, *p* = 0.779). (C) The probability of successfully moving downslope was also similar for both leap and roll behaviors (Fisher’s Exact test, df = 1, n = 51, *p* = 0.497). See text for additional details.

When placed with the body’s long axis perpendicular (at 90°) to the slope, mosquitofish are much more likely to produce a roll than a leap (Fisher’s Exact test, df = 1, n = 51, *p* = 0.00002; Figure 6A). In contrast, when mosquitofish are placed with the body’s long axis parallel to the slope, rolls and leaps occur at approximately the same frequency (Figure 6A). The probability of successfully moving downslope (Figure 6B) is similar from either body orientation (Fisher’s Exact test, df = 1, n = 53, *p* = 0.779) and is also similar (Figure 6C) for both movement classes (Fisher’s Exact test, df = 1, n = 51, *p* = 0.497). However, when all responses are considered together, fish were more likely to reach the bottom of the arena than not (33 successes vs. 20 failures); this pattern is significantly different from the null hypothesis of equal numbers of success and failures under a one-tailed *G*-test (*G* = 3.22, df = 1, n = 53, *p* = 0.073).

**Figure 7:**
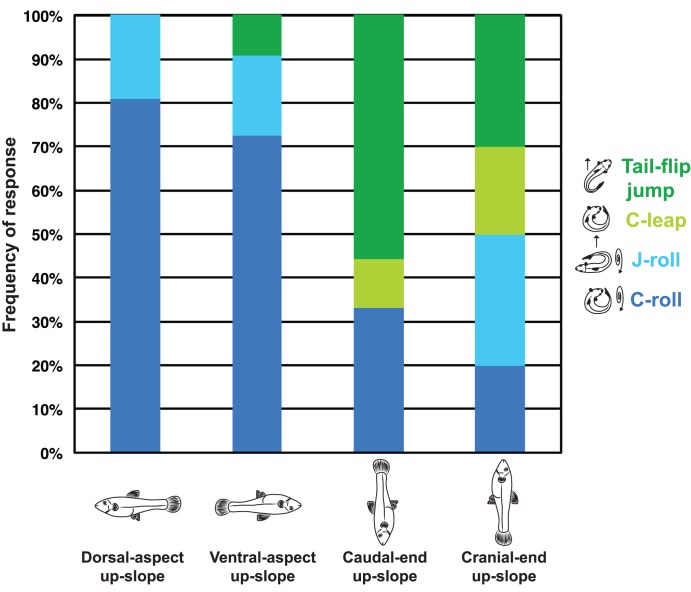
Mosquitofish (*Gambusia affinis*) responded similarly to being stranded in ventral-aspect up-slope (n = 11) and dorsal-aspect up-slope (n = 21) body positions, but produced different behaviors in response to being stranded in cranial-end up-slope vs. caudal-end up-slope body positions. When stranded cranial-end up-slope (n = 9), mosquitofish most often produced tail-flip jumps. When mosquitofish were stranded caudal-end up-slope (n = 10), all four behaviors were equally likely to occur (that is, each behavior occurred ∼25% of the time).

When mosquitofish were stranded with the long axis of their body parallel to the slope they appeared to be less likely to deploy a particular class of movement, relative to fish stranded with their bodies at 90° to the slope (Figure 6A). However, when the subcategories of body position (Figure 1) and movement type (Figure 3) are considered, additional patterns emerge (Figure 7). Tail-flip jumps appear to be favored by fish placed with their heads near the apex of the slope: mosquitofish placed in a cranial-end up-slope position produced tail-flip jumps five out of nine times (>50% of the time). A tail-flip jump will accelerate the head and anterior body over the tail and ultimately launch the fish into a caudually-oriented ballistic flight path [5]. Thus, a tail-flip jump enables a fish to move rapidly down-slope when it is positioned with its head toward the apex of the slope (see Video S1). In contrast, mosquitofish individuals stranded with the caudal peduncle (tail fin) at the top of the slope were equally likely to produce any one of the four possible behavioral responses (i.e., each of the four possible behaviors was produced approximately 25% of the time; Figure 7); individual mosquitofish appeared to arbitrarily select from among four behavioral options in response to this particular environmental challenge. Caudal-end up-slope appears to be a very difficult position for a fish to dislodge itself from, perhaps because a fish cannot readily induce a tumbling behavior about the long axis of the body and a tail-flip jump would initially move the fish upslope, rather than downslope. Thus, for a fish that finds itself in the caudal-end up-slope position, there may be no obvious behavioral solution for returning to the water.

### Downslope Trajectory

**Figure 8:**
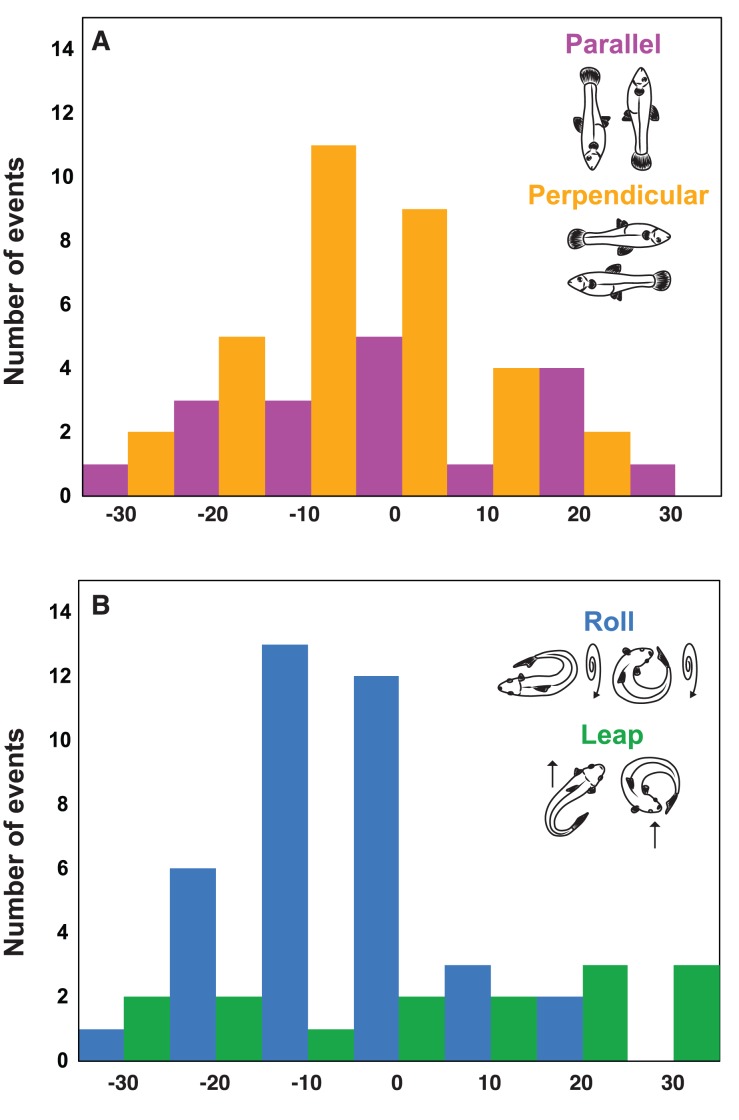
Mosquitofish (*Gambusia affinis*) placed in both perpendicular-to-the-slope (n = 33) and parallel-to-the-slope (n = 18) body orientations moved downslope within the filming arena to produce a mean response trajectory of ∼0° (upper panel). However, although both movement classes (leaps vs. rolls) performed in response to stranding generated similar mean response trajectories, leaps (n = 12) were characterized by a larger variance in movement trajectory (lower panel). Rolls (n = 39) were characterized by a smaller variance in movement trajectory and roll-type behaviors were more likely to move a mosquitofish directly down the center of the arena (lower panel); see text for additional details.

For both initial body orientations (parallel vs. perpendicular) and both movement classes (leaps vs. rolls), the mean trajectory of movement downslope (measured here as response trajectory) was approximately 0° (Figure 8). When perpendicular (trajectory mean ± SEM of ^+^4±4°) and parallel (^−^1±2°) body orientations were compared, the data were normally distributed (for perpendicular orientation, Shapiro-Wilk statistic = 0.971, n = 33, *p* = 0.498; for parallel orientation, Shapiro-Wilk statistic = 0.963, n = 18, *p* = 0.663), variances were homogenous (Levene statistic = 3.9, *p* = 0.54), and there was no significant difference in movement trajectory between the two body orientations (Welch statistic = 1.378, *p* = 0.251). When leaps (response trajectory mean ± SEM of ^+^5±6°) were compared to rolls (^−^1±2°), data were normally distributed (for leaps, Shapiro-Wilk statistic = 0.959, n = 11, *p* = 0.764; for rolls, Shapiro-Wilk statistic = 0.961, n = 38, *p* = 0.201) and, although the variances were heterogenous (Levene statistic = 9.55, *p* = 0.003), there was no difference in mean response between movement classes (Welch statistic = 1.491, *p* = 0.246). However, rolls tended to move fish directly down the experimental arena (the response trajectories clustered around 0°), whereas leaps generated trajectories distributed across possible outcomes and more often produced laterally oriented trajectories (Figure 8).

## Discussion

When mosquitofish are stranded such that their long-axis is perpendicular (90°) to the slope they consistently favor a roll-type behavior. The prevalence of roll-type behaviors in response to stranding was initially somewhat surprising because cyprinodontiform fishes have been observed performing tail-flip jumps (a type of leap) in the wild for a century or more [2], [3]. Yet, to the best of our knowledge, stranded fish in the wild have never been reported to produce roll-type behaviors. However, we suggest that rolls are favored over tail-flip jumps and other types of leaping in this situation for two reasons.

First, rolls are more likely than leaps to generate direct downslope movement. Evidence for this is provided by data clustered around 0° (directly downslope) for response trajectories that result from rolling. Leaps, in contrast, produce variable trajectories that are more likely to result in laterally-oriented movement. In the wild, response trajectories that displace a fish laterally might be less likely to facilitate a safe return to the water. Second, because a fish’s body remains in contact with the substrate throughout the behavior, rolls are energetically less expensive than leaps. We can infer an increased energetic cost of leaping relative to rolling because the mechanical work performed by a fish (or any organism) is a function of mass multiplied by the force of gravity and the height reached during the behavior. Thus, during a leap, the fish performs mechanical work to launch its mass into the air to increase its height above the substrate. During a roll, however, the fish never rises above the substrate; instead, stored potential energy is converted back into kinetic energy, enabling the fish to tumble downhill. Thus, at least for a steeply pitched, relatively smooth slope, rolling may be both more accurate and less energetically expensive than leaping.

The distribution of locomotor behaviors relative to body orientation displayed by mosquitofish stranded on a slope is clearly nonrandom. This “matching” of behavior to body orientation suggests that mosquitofish have the capacity to determine their body position relative to a slope and ascertain which direction is ‘downslope’ – which in most natural situations would be the location of the nearest body of water. This raises the question: *how* do stranded fish determine their position on a slope?

Visual cues could potentially assist a stranded fish in locating the water. However, visual cues (such as the water or the horizon) could be difficult for a stranded fish to interpret, given the evolutionary modifications to the aquatic vertebrate eye. Fish eyes have thick lenses that serve to accommodate the relatively high refractive index of water; because of this, most species are unable to focus images on the retina when light passes through air, which has a lower refractive index [8]. In addition, it is not clear what visual clues a fish would respond to in the artificial environment of a laboratory, where there is no natural horizon and no reflective pool of water nearby.

It seems probable that stranded fish employ the otolith-vestibular system, the mechanism used by fishes and other vertebrates to detect their orientation relative to gravity [9], to determine their position on a slope. Otoliths are dense crystalline structures that reside within the fluid-filled semicircular canals of all vertebrates [10], and similar structures are also present in some invertebrates [11]. The mineral composition of the otoliths varies among vertebrate groups [12]; in actinopteryigian fishes (the bony fishes, including the mosquitofish considered here), otoliths are endogenously produced from calcium and aragonite precursors. Because otoliths are always denser than the surrounding medium, they are negatively buoyant and rest on a bed of sensory hair cells. Movement of the otoliths in response to gravity (or acceleration) causes the hair cells in the bed to be deflected away from their resting position. Depending upon the direction and intensity of the deflection, the hair cells are excited or inhibited, and they trigger an increased or decreased rate of signaling to the central nervous system [13]. There are three pairs of otoliths in the vertebrate vestibular system, and each otolith pair is located within one of three semicircular canals that project into the X, Y, and Z planes of a Cartesian coordinate system. Based upon the pattern of excitation or inhibition of the hair cells from the ototliths, a fish (or any vertebrate) can determine the orientation of its head relative to the pull of gravity [14]; in fishes the head is simply an extension of the axial skeleton, thus the otolith-vestibular system provides information to a fish about the orientation of the entire axial body.

It appears that the otolith-vestibular system functions to allow a stranded mosquitofish to detect its body orientation when it is on land. On one hand, this is unsurprising because the otolith-vestibular system is ubiquitous among vertebrates. On the other hand, actinopterygian fishes possess a number of unusual and derived aspects of this system that are thought to be specializations for life in an aquatic medium [12]. For example, the utricle (one of the two otolith organs) is positioned at the apex of the horizontal and anterior vertical semicircular canals, where it will be affected by cranial movements in more than one dimension [12]. Such features could possibly reduce the fish’s ability to accurately detect body orientation when on land – which, for a fish out of water, represents a predominantly two-dimensional environment. Based on our observations, mosquitofish sensory systems appear to function sufficiently to allow stranded individuals to ascertain their position relative to a steep slope. However, it remains to be determined if the otolith-vestibular system in fishes that regularly find themselves out of water is modified when compared to species that never emerge onto land.

## Conclusions

Stranded mosquitofish typically do not immediately move in response to being stranded on a steep slope. Instead, they remain motionless for up to several minutes before they move the axial body. By staying immobile in the wild, it is possible that stranded fish enhance their ability to remain cryptic and undetected by pursing predators, although it is also possible that a stranded fish is simply trying to make sense of its body position relative to a novel environment and determine the appropriate behavioral response. When mosquitofish do move, they employ lateral contractions of the axial body to produce behaviors that propel them rapidly downslope – in the direction where the water would be located in most natural environments. In contrast with anecdotal accounts of fish “floundering” when out of water, the locomotor movements produced by mosquitofish in response to stranding represent distinct functional categories that are characterized by stereotypical movement patterns. These behaviors are quite effective in moving the fish rapidly in what is presumed to be the “desired” direction of travel.

In addition, it is clear that mosquitofish can sense their body orientation relative to the slope and alter their locomotor behavior accordingly. This is demonstrated by the fact that some behaviors are much more likely to be deployed in one body orientation than another. For example, rolls are almost always initiated when mosquitofish are stranded with the long axis of their body perpendicular (at 90°) to the slope. Because fish can convert stored potential energy into kinetic energy to tumble directly downslope, rolling is an energetically inexpensive and effective way to move back into the water from a cross-slope body orientation.

In contrast, leaps are more likely to be produced when a fish is stranded with its head up-slope. Although leaping requires additional mechanical work to elevate the fish’s mass against the forces of gravity, it can quickly move an individual downhill if the body is oriented in an appropriate position. We propose that mosquitofish modulate their behavior according to body orientation on a slope because each behavior provides a particular advantage when performed from a given starting position. By varying their response according to body orientation, a fish may increase its probability of a safe return to the water, relative to the energy it expends.

## Supporting Information

Appendix S1 **Summary table of data collected for a series of laboratory trials conducted using wild-caught mosquitofish (** ***Gambusia affinis*** **).** During these trials, 53 individual mosquitofish were manually stranded on a 30° artificial slope and their responses to stranding were recorded and quantified using digital video. See Methods for a complete description of the experimental protocol and an explanation of how each response variable was characterized and quantified.
(DOC)

Video S1 **Female mosquitofish #20 produced a tail-flip jump (a type of leap) in response to being stranded on a 30° slope with her cranial aspect oriented up-slope.** See Methods for a description of the experimental protocol and an explanation of how observed behavioral responses were categorized.
(MOV)

Video S2 **Female mosquitofish #36 produced a C-leap in response to being stranded on a 30° slope with her caudal aspect oriented up-slope.** See Methods for a description of the experimental protocol and an explanation of how observed behavioral responses were categorized.
(MOV)

Video S3 **Female mosquitofish #15 produced a C-roll in response to being stranded on a 30° slope with her dorsal aspect oriented up-slope.** See Methods for a description of the experimental protocol and an explanation of how observed behavioral responses were categorized.
(MOV)

Video S4 **Male mosquitofish #4 produced a J-roll in response to being stranded on a 30° slope with his dorsal aspect oriented up-slope.** See Methods for a description of the experimental protocol and an explanation of how observed behavioral responses were categorized.
(MOV)
