# Supplementary material for: Heads or Tails: Do Stranded Fish (Mosquitofish, Gambusia affinis) Know Where They Are on a Slope and How to Return to the Water?
Source: PLoS One. 2014 Aug 27;9(8):e104569. doi: 10.1371/journal.pone.0104569 (PMC4146521; doi:10.1371/journal.pone.0104569)
Supplement: Appendix S1 — Summary table of data collected for a series of laboratory trials conducted using wild-caught mosquitofish ( Gambusia affinis ). During these trials, 53 individual mosquitofish were manually stranded on a 30° artificial slope and their responses to stranding were recorded and quantified using digital video. See Methods for a complete description of the experimental protocol and an explanation of how each response variable was characterized and quantified. (DOC) [file pone.0104569.s001.doc]

**Appendix 1**

Fish = ID number

Sex = male (m) vs. female (f)

Position = cranial end up, caudal end up, dorsal aspect up, or ventral aspect up (where “up” indicates orientation toward the top of the slope)

Orientation = parallel vs. perpendicular (orientation of the long axis of the body, relative to the slope)

Movement type = Tail-flip jump, C-leap, J-roll, or C-roll

Movement class = leap or roll

Outcome = success or failure

Trajectory = angle of movement down the arena in degrees (°), where 0° indicates a direct vertical path down the arena

Latency time = time to initiate movement after being placed on substrate (s)

Landing time = duration of the movement, measured as time elapsed from when fish started moving to when fish stopped moving (s)

| Fish | Sex | Mass (g) | Standard Length (mm) | Total Length (mm) | Position | Orientation | Movement Type | Movement Class | Outcome | Trajectory (°) | Latency time (s) | Landing time (s) |
| --- | --- | --- | --- | --- | --- | --- | --- | --- | --- | --- | --- | --- |
| 1 | F | 0.921 | 35.24 | 41.52 | Cranial end up | Parallel | Tail-flip jump | Leap | Success | 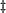 | 31.98 | 0.17 |
| 2 | F | 2.117 | 46.55 | 55.78 | Caudal end up | Parallel | C-leap | Leap | Success | 40 | 00.28 | 0.73 |
| 3 | F | 0.532 | 30.08 | 34.72 | Caudal end up | Parallel | C-roll | Roll | Failure | 3 | 29.18 | 0.78 |
| 4 | F | 0.819 | 32.04 | 38.70 | Dorsal aspect up | Perpendicular | C-roll | Roll | Failure | 24 | 55.52 | 1.27 |
| 5 | F | 1.502 | 41.92 | 50.79 | Caudal end up | Parallel | C-roll | Roll | Failure | 2 | 33.95 | 1.25 |
| 6 | F | 0.775 | 33.14 | 37.98 | Ventral aspect up | Perpendicular | C-roll | Roll | Success | 17 | 34.32 | 0.92 |
| 7 | F | 2.600 | 48.50 | 58.94 | Cranial end up | Parallel | C-roll | Roll | Success | 20 | 16.42 | 0.83 |
| 8 | F | 1.612 | 40.83 | 49.84 | Caudal end up | Parallel | * | * | Success | 17 | 42.50 | 0.65 |
| 9 | F | 0.829 | 33.36 | 38.96 | Ventral aspect up | Perpendicular | C-roll | Roll | Success | 16 | 31.42 | 1.15 |
| 10 | F | 1.087 | 39.15 | 47.27 | Cranial end up | Parallel | C-leap | Leap | Failure | 24 | 16.65 | 0.28 |
| 11 | F | 1.461 | 40.51 | 48.40 | Ventral aspect up | Perpendicular | C-roll | Roll | Success | 2 | 17.15 | 0.63 |
| 12 | F | 0.204 | 22.19 | 26.54 | Dorsal aspect up | Perpendicular | C-roll | Roll | Failure | -12 | 32.15 | 0.58 |
| 13 | F | 1.342 | 38.53 | 46.91 | Cranial end up | Parallel | C-roll | Roll | Failure | -6 | 37.50 | 1.03 |
| 14 | F | 0.503 | 29.78 | 35.33 | Cranial end up | Parallel | Tail-flip jump | Leap | Success | ‡ | 28.08 | 0.77 |
| 15 | F | 1.253 | 36.89 | 45.22 | Dorsal aspect up | Perpendicular | C-roll | Roll | Success | -21 | 49.85 | 0.52 |
| 16 | F | 0.836 | 35.10 | 41.29 | Ventral aspect up | Perpendicular | J-roll | Roll | Success | -22 | 63.72 | 1.00 |
| 17 | F | 2.472 | 48.78 | 58.21 | Dorsal aspect up | Perpendicular | C-roll | Roll | Failure | -8 | 35.83 | 0.48 |
| 18 | F | 0.450 | 26.91 | 32.37 | Cranial end up | Parallel | Tail-flip jump | Leap | Success | -15 | 25.10 | 0.72 |
| 19 | F | 0.979 | 35.74 | 43.57 | Ventral aspect up | Perpendicular | C-roll | Roll | Success | -3 | 24.03 | 0.77 |
| 20 | F | 0.204 | 23.70 | 29.96 | Cranial end up | Parallel | Tail-flip jump | Leap | Success | 30 | 33.58 | 0.40 |
| 21 | F | 1.304 | 38.13 | 46.40 | Ventral aspect up | Perpendicular | C-roll | Roll | Success | -15 | 15.50 | 0.60 |
| 22 | F | 0.834 | 34.15 | 42.24 | Caudal end up | Parallel | J-roll | Roll | Success | -8 | 16.17 | 0.98 |
| 23 | F | 0.743 | 30.06 | 37.62 | Dorsal aspect up | Perpendicular | C-roll | Roll | Failure | 9 | 38.18 | 0.40 |
| 24 | F | 0.262 | 24.25 | 30.07 | Ventral aspect up | Perpendicular | Tail-flip jump | Leap | Failure | -19 | 23.05 | 0.65 |
| 25 | F | 1.430 | 39.17 | 48.05 | Dorsal aspect up | Perpendicular | C-roll | Roll | Success | -6 | 47.80 | 2.22 |
| 26 | F | 1.213 | 36.89 | 44.65 | Caudal end up | Parallel | J-roll | Roll | Success | 3 | 45.12 | 0.70 |
| 27 | F | 0.326 | 30.71 | 25.22 | Dorsal aspect up | Perpendicular | C-roll | Roll | Success | 3 | 46.85 | 1.15 |
| 28 | F | 0.789 | 30.08 | 38.60 | Ventral aspect up | Perpendicular | C-roll | Roll | Success | -8 | 32.32 | 0.58 |
| 29 | F | 0.682 | 30.29 | 36.78 | Cranial end up | Parallel | C-roll | Roll | Failure | -18 | 29.98 | 2.12 |
| 30 | F | 1.218 | 39.17 | 46.95 | Dorsal aspect up | Perpendicular | C-roll | Roll | Success | -14 | 58.13 | 0.58 |
| 31 | F | 1.830 | 44.35 | 52.01 | Dorsal aspect up | Perpendicular | C-roll | Roll | Failure | -5 | 40.02 | 0.58 |
| 32 | F | 1.319 | 42.94 | 47.47 | Caudal end up | Parallel | J-roll | Roll | Failure | -16 | 188.25 | 1.05 |
| 33 | F | 1.390 | 39.46 | 48.65 | Ventral aspect up | Perpendicular | J-roll | Roll | Success | -1 | 10.35 | 0.82 |
| 34 | F | 0.238 | 22.79 | 27.73 | Caudal end up | Parallel | Tail-flip jump | Leap | Failure | 5 | 18.45 | 0.38 |
| 35 | F | 0.968 | 38.73 | 38.33 | Dorsal aspect up | Perpendicular | * | * | Failure | 11 | 43.83 | 0.68 |
| 36 | F | 2.018 | 43.39 | 53.68 | Caudal end up | Parallel | C-leap | Leap | Success | -1 | 31.82 | 0.70 |
| 37 | F | 2.113 | 45.69 | 55.30 | Dorsal aspect up | Perpendicular | C-roll | Roll | Failure | -9 | 55.93 | 0.70 |
| 38 | F | 0.262 | 24.74 | 31.23 | Caudal end up | Parallel | Tail-flip jump | Leap | Success | 1 | 26.57 | 0.53 |
| 39 | F | 0.537 | 29.71 | 35.70 | Ventral aspect up | Perpendicular | C-roll | Roll | Failure | -1 | 25.83 | 1.62 |
| 40 | F | 0.848 | 32.82 | 38.62 | Cranial end up | Parallel | Tail-flip jump | Leap | Success | -24 | 20.33 | 0.78 |
| 41 | F | 2.190 | 44.64 | 52.77 | Caudal end up | Parallel | Tail-flip jump | Leap | Success | 23 | 69.97 | 1.12 |
| 42 | F | 1.645 | 40.81 | 48.63 | Ventral aspect up | Perpendicular | C-roll | Roll | Success | ‡ | 52.30 | 1.17 |
| M1 | M | 0.295 | 22.65 | 28.44 | Dorsal aspect up | Perpendicular | C-roll | Roll | Failure | 40 | 19.67 | 0.43 |
| M2 | M | 0.209 | 23.79 | 30.46 | Dorsal aspect up | Perpendicular | J-roll | Roll | Failure | 3 | 83.05 | 0.57 |
| M3 | M | 0.220 | 23.67 | 28.50 | Dorsal aspect up | Perpendicular | C-roll | Roll | Success | 24 | 114.37 | 0.85 |
| M4 | M | 0.263 | 26.42 | 30.58 | Dorsal aspect up | Perpendicular | J-roll | Roll | Failure | 2 | 59.05 | 0.43 |
| M5 | M | 0.257 | 21.00 | 26.71 | Dorsal aspect up | Perpendicular | J-roll | Roll | Failure | 18 | 57.08 | 0.52 |
| M6 | M | 0.270 | 24.77 | 30.02 | Dorsal aspect up | Perpendicular | J-roll | Roll | Success | 20 | 72.30 | 0.47 |
| M7 | M | 0.133 | 24.87 | 31.80 | Dorsal aspect up | Perpendicular | C-roll † | Roll | Success | 17 | 68.17 | 0.40 |
| M8 | M | 0.246 | 25.80 | 30.49 | Dorsal aspect up | Perpendicular | C-roll | Roll | Success | 16 | 66.90 | 0.48 |
| M9 | M | 0.251 | 25.02 | 29.90 | Dorsal aspect up | Perpendicular | C-roll | Roll | Success | 24 | 00.07 | 0.83 |
| M10 | M | 0.265 | 23.88 | 29.54 | Dorsal aspect up | Perpendicular | C-roll | Roll | Success | 2 | 77.08 | 1.27 |
| M11 | M | 0.258 | 22.55 | 27.60 | Dorsal aspect up | Perpendicular | C-roll | Roll | Success | -12 | 49.37 | 0.95 |

* Behavior could not be categorized.

† C-roll in which the initial body bend was toward the substrate.

‡ Fish jumped beyond edge of apparatus and trajectory of response could not be measured.
